# A milk‐line sampling system to detect foodborne pathogens: A field case investigation from the United States and Argentina

**DOI:** 10.1002/vms3.484

**Published:** 2021-05-16

**Authors:** Florencia Farcey, Julian Bartolome, Carlos Roeschmann, Javier Chaves, Hemant K. Naikare, Pedro Melendez

**Affiliations:** ^1^ College of Veterinary Medicine National University of La Pampa Santa Rosa Argentina; ^2^ College of Veterinary Medicine University of Chile Santiago Chile; ^3^ Tifton Veterinary Diagnostic and Investigational Laboratory College of Veterinary Medicine University of Georgia Tifton GA USA

**Keywords:** dairy cattle, foodborne pathogens, milk, sampling device

## Abstract

The objective of this short communication was to discuss two field case investigations to determine the usefulness of a milk‐line sampling device to detect bacteria either coming from a group of cows suffering from mastitis or from the milking line potentially contaminated with environmental bacteria. In Case 1, the in‐line sampling device was able to detect certain segments of the milk‐line contaminated with environmental bacteria, but not coming from the cows. In Case 2, 19 out of 25 pooled in‐line samples were in agreement with at least one of the individual sampled cows shedding either *Staphylococcus* or *Streptococcus spp.* or both, which accounted for 76% accordance between both methods. The in‐line system, although not perfect, provided a reliable method to detect individual cows shedding mastitis‐causing organisms. In conclusion, the milk‐line sampling device system was able to help identify foodborne pathogens. Regular monitoring of the microbial quality of milk through a milk‐line sampling device is recommended for groups of cows within the dairy herd to detect potential mastitis‐causing microorganisms. Furthermore, the sampling device was an effective tool to screen the efficacy of cleaning and disinfecting mechanisms of the milk lines to identify and control potential foodborne pathogens that are collected in the bulk tank.

## Graphical abstract

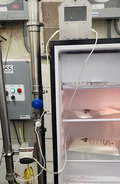

## 1 INTRODUCTION

The opportune detection and identification of the source of bacterial contamination of milk from lactating cows is essential to take preventive actions to decrease the risk of mastitis at the level of the herd and food poisoning in humans (Godden et al., 2002; Nagoette et al., 2019). Bacteria may come both from cows and their infected udders and from environmental saprophytic contamination of segments of the milk line before the milk reaches the bulk tank (Gonzalez et al., 1986; Smith et al., 1985).

QualiTru Sampling System (Oakdale, Minnesota 55,128, USA) is a sampling device that can be set up at any connection of the milking line from the parlour to the bulk tank. The system consists of a stainless steel port that can be connected to any junction of the milking line to obtain a representative milk sample from a group of cows or the entire milking herd. Milk‐line sampling may be particularly attractive to larger herds because it allows producers to monitor the performance of several different groups of cows within the herd (Godden et al., 2002). In addition, the system could be used to monitor any potential contamination from different sections of the milk line.

In this article, we report the usefulness of a milk‐line sampling device to detect bacteria either coming from a group of cows shedding mastitis‐causing microorganisms or from the milking line potentially contaminated with environmental bacteria. This report summarizes two field case investigations that occurred in a dairy herd in the United States (Case 1) and in Argentina (Case 2).

## 2 CASE DESCRIPTION

### 2.1 Case 1

This case occurred in a dairy farm from the south of Georgia, USA, which had a history of high Preliminary Incubation Count bacteria (>30,000 CFU) in the bulk tank milk. One group of 16 cows (#1–16) was individually sampled for an experienced veterinarian to obtain a composite of the four quarters right before milking. Before beginning milking, a line sampling device (QualiTru Sampling System, Oakdale, Minnesota 55,128, USA) was set up in the first junction of the milk‐line after leaving the parlour. A sterile septum was placed in the stainless steel port. Then, a sterile 18‐G needle with a line connected to a sterile bag was inserted in the septum. Before reaching the sterile bag, the line passed through a portable peristaltic pump that allowed a frequency flow sampling over the entire process run. The pump was set up for a volume of sampling of 60 ml of milk per minute.

Individual cow composite samples (all four quarters) and milk‐line pooled sample were refrigerated and then transported directly to a Veterinary Diagnostic and Investigational Laboratory. After arrival, samples were cultured for aerobic bacterial isolation and *Mycoplasma sp.* using standard approved methodologies. The group of cows sampled showed that cow #1, 2, 3, 4, 6, 7, 8 and #10–16 were negative to bacteria isolation. Cow #5 was positive to coagulase negative *Staphylococcus* (CNS), and cow #9 was positive for *Prototheca*. However, milk‐line pooled sample was positive to non‐hemolytic *Streptococcus*, *Klebsiella pneumoniae* ssp *pneumoniae*, *Acinetobacter* sp, *Klebsiella* ssp *ozaenae*, *Enterococcus durans* and *Streptococcus bovis*. Interestingly, none of the bacteria isolated from the sampling device were present in the individual cow cultures, suggesting that the milk‐line was highly contaminated with biological material from milk and several environmental bacteria. As was expected, most of the cow samples (except #5 and 9) were negative, indicating a good sampling protocol carried out by the experienced veterinarian. This finding highlights the efficacy of the in‐line sampling device to detect certain segments of the milk‐line potentially contaminated with environmental bacteria. These bacteria from the milk‐line may turn into contaminants of the bulk tank milk which can become foodborne pathogens for human consumption.

### 2.2 Case 2

This case occurred in a large dairy farm in the pampas of Argentina. Cows were milked in seven different groups of 170 cows according to days in milk and milk production. The farm had a history of high somatic cells count (>350,000 cells/ml) and high incidence of clinical cases of mastitis. Even though the majority of cases were environmental based on previous bacteriological culture data, cows with *Staphylococcus aureus* and *Mycoplasma spp* infection were detected. The objective was to test the ability of an in‐line sampling system to detect *Staphylococcus aureus* in a group of cows whenever a cow was positive within that particular group. Since sampling and culturing all cows of the entire herd was not economically feasible, only a select few cows with high somatic cell counts or with clinical mastitis within a group were selected to be individually sampled and compared to the findings with the in‐line sample from that group of cows. Sampling was performed the same way as described in Case 1 and individual cow composite (four quarters) samples (12 cows per line) and milk in‐line pooled samples were refrigerated and then transported directly to an accredited laboratory to perform standard bacteriological procedures for isolation and identification of mastitis pathogens. Twenty‐five pooled samples using the in‐line sampling device were obtained and procedures at the laboratory included a modified NMC 2004 protocol (National Mastitis Council, 2004). Each in‐line pooled sample was the reflection of 24 cows milked per size in the parlour. For *Staphylococcus aureus* and *Staphylococcus spp* detection, samples were plated on Vogel Johnson agar and isolated colonies were catalase positive, mannitol fermenters (48 hr) and screened by coagulase test. For *Streptococcus agalactiae* and other *Streptococcus spp* detection, samples were plated on Edward agar and isolated colonies were catalase negative and esculin positive. The esculin negative isolates were subjected to the CAMP test to differentiate *Streptococcus agalactiae*. In Table 1, descriptive data of in‐line milk cultures and cow composite cultures are shown. For example, sample #1 from the in‐line pooled sample was positive to *Staphylococcus aureus*; however, from individual animals, four cow samples had contaminated bacteria, one cow sample was positive to *Prototheca*, and one cow was positive to *Streptococcus uberis*. In this case, there was no agreement between the pooled sample and individual samples, because none of the bacteria isolated from the individual sampling were present in the in‐line sample. Sample #2 from the in‐line pooled sample was positive to *Staphylococcus aureus*. From individual animals, two cows were positive to *Staphylococcus aureus*, one cow had contaminated bacteria, and two cows were positive to *Streptococcus spp*. In this case, there was agreement between the in‐line sample and individual sampling, because two cows that were shedding *Staphylococcus aureus* were detected in the in‐line sample. Notably, 19 out of the 25 pooled in‐line samples were in agreement with at least one of the individual samples, which accounted for 76% accordance between the two methods. In this sense, the in‐line system, although not perfect, provides a reliable mechanism to detect individual cows shedding either *Staphylococcus* or *Streptococcus spp.* or both.

**TABLE 1:** Descriptive data of in‐line milk microbial cultures and cow composite milk microbial cultures in Argentina

Date and sample #		Bacteria from milk‐line device	Bacteria detected from individual cultures	Agreement with	
1/21/2020	1	*S. aureus*	4 contaminated, 1 *Prototheca*, 1 *S. uberis*	No	*Strep/Proto*
	2	*S. aureus*	2 *S. aureus*, 1 contaminated, 2 *Strep*	Yes	*Staph*
	3	*S. aureus*	3 *S. aureus*, 2 contaminated, 2 *St. uberis*, 1 *Corynebacterium*	Yes	*Staph*
	4	*Streptococcus sp*.	2 *Strep*, 1 contaminated	Yes	*Strep*
	5	*Streptococcus sp*	1 *Strep*, 2 contaminated, 2 *E. coli*	Yes	*Strep*
					
2/14/2020	6	*Strep/E.coli*	1 *Strep*, 6 *Staph*, 1 *E coli*, 1 *Bacillus*, 1 *Corynebacterium*	Yes	*Strep*
	7	*Strep/E.coli*	2 *Staph*, 1 *Corynebacterium*, 2 *Strep*, 1 *Serratia*	Yes	*Strep*
	8	*Strep/E. coli/Staph*	1 *Staph*, 1 *Strep*, 1 contaminated, 2 *Corynebactacterium*	Yes	*Strep/Staph*
	9	*Strep/E.coli/Staph*	1 *Staph*	Yes	*Staph*
	10	*Strep/E. coli*	3 *Staph*, 2 *Strep*	Yes	*Strep*
	11	*Strep/E.coli/Staph*	1 *Strep*, 1 contaminated	Yes	*Strep*
	12	*Strep/E.coli/Staph*	1 *Staph*, 1 *Strep*	Yes	*Strep/Staph*
					
3/13/2020	13	*Strep/E.coli/Staph*	3 *Strep*, 1 *Staph*, 1 *Corynebacterium*	Yes	*Strep/Staph*
	14	*Strep/E.coli*	None positive	No	
	15	*Strep/E.coli*	3 *Staph,* 1 contaminated	No	*Staph*
	16	*Strep/E.coli*	None positive	No	
	17	*Strep/E.coli/Staph*	2 *Staph*, 1 *E. coli*	Yes	*Staph/E.coli*
	18	*Strep/E.coli/Staph*	1 *Strep*, 1 contaminated	Yes	*Strep*
	19	*Strep/E.coli/Staph*	1 *Strep*, 1 *Corynebacterium*, 1 *Bacillus*, 1 contaminated	Yes	*Strep*
					
4/28/2020	20	*Strep/Staph*	2 contaminated, 2 *Nocardia*, 2 *Staph*, 1 *Strep*	Yes	*Strep/Staph*
	21	*Strep/Staph*	2 *Staph*, 1 *Klebsiella*, 1 *Pasteurella*	Yes	*Staph*
	22	*Strep/Staph*	1 *Staph*, 1 contaminated, 1 *Enterecoccus*, 2 *Prototheca*, 1 *Pseudomonas*	Yes	*Staph*
	23	*Strep/Staph*	1 coliform, 1 *Corynebacterium*, 2 *Klebsiella*	No	*E.coli*
	24	*Strep/Staph*	2 *Staph*, 1 *Nocardia*, 1 contaminated	Yes	*Staph*
	25	*Strep/Staph*	1 *Prototheca*, 1 *Staph*, 2 *Strep*	Yes	*Strep/Staph*

Abbreviations: *Staph*, *Staphylococcus species*;*Strep*, *Streptococcus species*.

In conclusion, in both cases, milk‐line sampling device system was able to help identify foodborne pathogens. Regular monitoring of the microbial quality of milk through a milk‐line sampling device is recommended for groups of cows within the dairy herd to detect potential mastitis‐causing microorganisms. Furthermore, the sampling device is an effective tool to screen the efficacy of cleaning and disinfecting mechanisms of the milk lines to identify and control the potential foodborne pathogens that are collected in the bulk tank.

## ETHICS STATEMENT

The authors confirm they followed the ethics policies of the journal and the University of Georgia.

## CONFLICT OF INTEREST

The authors have no conflict of interest to declare.

## AUTHOR CONTRIBUTIONS

FF participated in milk sample collection and bacteria culturing at the US and Argentina dairy. JB and JC conducted farm visits, sampling and culture interpretation in Argentina. CR participated in milk sampling at the US dairy. HN participated in milk sample culturing, interpretation of culture results and manuscript writing. PM participated in milk sampling at the US dairy, and manuscript writing. All authors read and approved the final manuscript.

### PEER REVIEW

The peer review history for this article is available at https://publons.com/publon/10.1002/vms3.484.
